# The association between lean mass and bone mineral content in the high disease activity group of adult patients with juvenile idiopathic arthritis

**DOI:** 10.1186/1471-2474-15-51

**Published:** 2014-02-21

**Authors:** Kristyna Brabnikova Maresova, Katerina Jarosova, Karel Pavelka, Jan J Stepan

**Affiliations:** 1 Institute of Rheumatology, Na Slupi 4, 128 50 Prague, Czech Republic; 2 First Faculty of Medicine, Charles University in Prague, Prague, Czech Republic

**Keywords:** JIA in adults, Disease activity, DAS 28, Body composition, Lean mass, Bone mineral density, Bone mineral content, Glucocorticoids

## Abstract

**Background:**

The study is aimed to evaluate body composition and bone status in adolescent and adult patients with active juvenile idiopathic arthritis (JIA) untreated with tumor necrosis factor alpha inhibitors.

**Methods:**

Adult patients (12 male and 19 female) with active JIA and 84 healthy age- and gender- matched controls were enrolled into the study. Body composition (tissue mass in grams, lean mass, fat mass and bone mineral content as a fraction of tissue mass) and areal bone mineral density parameters (aBMD) at the lumbar spine, proximal femur, femoral neck, distal radius and total body were assessed using dual energy x-ray absorptiometry (DXA), and correlated with clinical characteristics of the disease and physical performance tests. Disease activity was assessed using high-sensitivity C-reactive protein (hsCRP) and disease activity score 28 (DAS 28). Differences between the groups were tested by *t*-test, and One-way ANOVA. Correlations were assessed using the Pearson correlation coefficients and multiple linear regression analysis. Significances were counted at the 0.05 level.

**Results:**

In patients with clinically active JIA (DAS 28, 6.36 ± 0.64, hsCRP, 18.36 ± 16.95 mg/l), aBMD at all measured sites, bone mineral content (BMC) and lean mass were reduced, and fat mass was increased as compared with healthy controls. Significant negative correlations were observed between BMC and disease duration, use of glucocorticoids (GCs), and fat mass, respectively. A positive correlation was found between BMC and lean mass, and between the body fat fraction and the use of GCs. Using multiple linear regression analysis, lean mass was the only significant predictor of BMC of total body both in men and women, and of BMC of legs (only in men). Lean mass was also the only predicting factor of total proximal femur BMD and femoral neck BMD. No significant correlations have been determined among the body composition parameters and DAS 28 or hsCRP endpoints.

**Conclusions:**

In adult patients with long-term active JIA, lean mass was the main determining factor of total body and leg BMC, and total proximal femur and femoral neck aBMD.

## Background

Juvenile idiopathic arthritis (JIA) is a systemic connective tissue disease with onset before age 16. This autoimmune inflammatory disease is associated with potential focal and systemic bone loss, and consequently with decreased bone mineral density (BMD) [1,2], and a lifetime increased risk of fractures [3]. The pathophysiology of bone loss involves especially deleterious effects of the pro-inflammatory cytokines produced by the synovial membrane and also glucocorticoid (GC) treatment [4,5]. Both the excessive bone resorption [5] and decreased bone formation and osteoblast function are responsible for bone loss in patients with JIA [6,7]. Reduced BMD is observed at all sites of the skeleton in children, adolescents as well as in adults with JIA. In the cross-sectional study, the low BMD in lumbar spine and hip was found in 42–52% of adult patients with JIA [8]. The total body and local growth retardation of children with JIA is well described [9]. In children and adolescents with JIA, biological treatment with tumor necrosis factor alpha (TNFα) blockers infliximab or etanercept is associated with a decrease in disease activity. A positive effect of the therapy on the skeleton was also documented [10].

Decrease in bone mass in JIA is also associated with muscle atrophy. A linear relationship was described between muscle cross-sectional area and bone mineral content (BMC) of radial diaphysis in healthy children and adolescents [11]. The bone-muscle unit plays an important role especially in the growing bones of children and adolescents. It is the muscle forces, not body weight, that load the load-bearing bones. Bones adapt their strength to maintain the strain caused by physiological loads close to a set point and the largest physiological loads are caused by muscle contractions [12], and muscle strength thus strongly influences postnatal bone strength [13]. In JIA, inflammation, low physical activity as well as the GC therapy may be responsible for muscular atrophy.

Therefore, the aim of the present study is to assess the association between disease activity, glucocorticoid therapy, and body composition in adolescent and adult patients with long-term severe JIA before the initiation of treatment with TNFα blockers. The results of this study have showed significant differences between adult patients with active JIA and healthy controls in aBMD and body composition. In JIA patients the lean mass was the main determining factor of BMC of total body and legs, and proximal femur and femoral neck aBMD.

## Methods

### Study design, participants

The study reports baseline data in 12 male and 19 female adult patients with active JIA before the initiation of treatment with TNFα blockers. According to the criteria of the Czech Rheumatology Society, the basic indication for therapy with TNFα inhibitors is an unsatisfactory response to therapy with one disease-modifying anti-rheumatic drug (DMARD) (preferably methotrexate, alternatively sulphasalazine or leflunomide). DMARD therapy before TNFα blockers initiation must be at least 3-6 months with adequate dosage (methotrexate dose 20-30 mg). The other basic condition is a disease activity score 28 (DAS 28) of at least ≥ 3.9 [14]. The lowest DAS 28 in our JIA group was 5.1.

The control sample of young men and women with no fracture was recruited by invitation in the same district of Prague. The volunteer group (100 subjects) was selected randomly from classmates, friends and acquaintances of JIA patients. From these invitations, three eligible age- and gender- matched control participants (only 2 control participants in 9 females) were selected for each JIA case. Wherever a precise match by the year of birth was not possible, the closest matching case was selected in most cases up to a maximum of a 2-year age difference (rarely up to a maximum of 6-year age difference). Thus, 84 controls were available from the volunteer group, and the age of the volunteers was matched for the age of patients.

The patients and controls were examined in the Institute of Rheumatology in Prague. All participants gave their written informed consent before enrollment. The study protocol and informed consent documents were prepared in compliance with the Declaration of Helsinki and approved by the local ethical review board. The study was approved by the Ethics Committee of the Institute of Rheumatology. The authors have complied with the World Medical Association Declaration of Helsinki regarding ethical conduct of research involving human subjects.

The subjects completed clinical examination, bone mineral density and body composition measurement and blood sampling. The type and duration of disease and previous therapy were recorded for each patient. A complete clinical history, including details of co-morbidity, detailed personal history of JIA, GC use (previous or ongoing, dosage, duration, and route of administration), fracture history (type and trauma), alcohol intake, smoking, height loss, family history of osteoporosis and hip fracture, and physical examination were assessed by the same physician. The control subjects were not supplemented with vitamin D and calcium. The JIA patients were supplemented with 1000 mg calcium and 800 IU vitamin D daily, for at least six months prior to evaluation. The body height was measured with a stadiometer and body weight with an accurate scale.

### Disease activity

Disease activity was assessed using high-sensitivity C-reactive protein (hsCRP) and DAS 28. hsCRP serum concentrations were measured using immunoturbidimetry. Inter-day coefficient for variation for hsCRP was 1.9%. DAS 28 was assessed using DAS 28 calculator including objective clinical, laboratory as well as subjective components. The components were the number of tender and swollen joints (from the total number of 28), erythrocyte sedimentation rate and patient global health (0 = best, 100 = worst).

### Bone densitometry

Dual energy x-ray absorptiometry (DXA, bone densitometer Prodigy, GE, U.S.A., Software 12.10.113) was used to measure aBMD at lumbar spine, total proximal femur, femoral neck, femur trochanter and distal radius. The short-term in-vivo precision errors for lumbar spine, total femur, femoral neck and distal radius BMD were 0.7%, 0.9%, 1.8% and 0.9%, respectively; the long-term precision error using the Hologic phantom was 0.31%. Daily scanning of a phantom showed an absence of machine drift during the study. aBMD was expressed in g/cm^2^ and in T-scores. Normative values provided by GE Prodigy were used for the determination of T-scores (comparison with an average bone density of young healthy adults of the same gender). In all subjects, the DXA was measured using the same instrument and technician in order to eliminate operator discrepancies, and it was assessed by the same physician.

The availability of DXA enables the precise measurement of body composition in terms of lean and fat mass and bone mineral content of the total body, trunk, legs and arms. In our study we calculated percentages for lean mass, fat mass and BMC evaluation. BMC, lean mass and fat mass were measured using whole-body absorptiometry software of the bone densitometer (Prodigy, GE, U.S.A.) and were expressed in grams. Percentages of BMC, lean mass and fat mass were calculated by dividing each absolute value by total mass. For instance percentage trunk fat was calculated by dividing trunk fat mass by total fat mass and was designated (%) trunk fat. A strong correlation between body weight and total body mass as measured by DXA (r = 0.98) was obtained in a preliminary study. The coefficients of variation of measurements of BMC, lean and fat mass were 0.9, 1.0 and 2.0%, respectively.

### Physical performance tests of lower limbs (legs)

The authors used 2 tests for muscle strength of lower limbs assessment. In chair rise test the goal was to get up from a chair and then to sit down as quickly as possible 5 times in a row (seconds); the lower the value, the better the result of lower extremity muscle strength. Walking speed was calculated based on the time needed to complete the required number of meters (meters per second); the higher the value, the better the result of lower extremity muscle strength.

### Statistical methods

Thirty one JIA patients and 84 age- and gender-matched control individuals were included in the analysis. Summary statistics including group size, mean, and SD were reported for each parameter. Differences between the groups were tested by *t*-test, and One-way ANOVA. Correlations were assessed using the Pearson correlation coefficients and multiple linear regression analysis. Significances were counted at the 0.05 level.

## Results

Summary statistics of the JIA patients and healthy control subjects are given in Table 1. The mean of JIA onset was at the age of 10.3 ± 4.9 years and disease duration was 14.6 ± 9.1 years. The JIA subtypes seen in patients were as follows: polyarticular in 16 patients (rheumatoid factor positive in 4, rheumatoid factor negative in 12), enthesitis-related arthritis in 9 patients, extended oligoarticular in 4 patients, psoriatic in 2 patients. Functional class I was determined in 18 patients, II in 4, III in 5, and IV in 4 patients. 6 patients were ANA positive. Cervical spine involvement was found in 7 patients. No patients suffered from vasculitis or lung impairment. In females, the menarche age was 13.1 ± 1.2 years. In JIA patients the value of Health Assessment Questionnaire was 0.97 ± 0.60 and EuroQol Questionnaire was 0.56 ± 0.27.

**Table 1:** Clinical characteristics of JIA patients and controls

	**JIA patients (n = 31)**	**Controls (n = 84)**	**p**
No of participants	31	84	
Male/female (No)	12/19	36/48	0.692
Age (years)	25.1 ± 6.1	23.8 ± 4.5	0.405
Anthropometric measures			
Age (years)	25.1 ± 6.1	23.8 ± 4.5	0.405
Height (cm)	170.5 ± 9.8	173.3 ± 9.3	0.145
Weight (kg)	68.0 ± 12.5	69.2 ± 12.5	0.884
BMI (kg/m^2^)	23.4 ± 3.9	22.9 ± 3.0	0.435
Clinical data			
Vertebral fractures (No)	5	0	<0.001
Non-vertebral fractures (No)	6	0	<0.001
Family hip fracture history (No)	0	0	
Smoking (No)	7	13	0.372
Alcohol abuse (No)	0	0	
Menarche (years)	13.1 ± 1.2	12.9 ± 1.1	0.994
Contraception in female (No)	11	23	0.462
Serum 25(OH)D (nmol/l)	65.9 ± 42.6	50 ± 28.5	0.029
Disease activity			
DAS 28	6.36 ± 0.64	-	
hsCRP (mg/l)	18.36 ± 16.95	1.40 ± 1.63	<0.001

Displayed are numbers or means ± SD. Clinical characteristics with zero value were not statistically calculated.

At the time of study recruitment, all of the patients were treated with DMARDs, 23 with methotrexate (mean dose of 16.8 ± 3.4 mg/week), 4 with leflunomide, 2 with sulphasalazine, 1 with sulphasalazine + hydroxychloroquine sulfate and 1 with cyclosporine A. Nine women and 3 men were current users of GCs, 10 patients with Prednisone, 2 patients with Medrol. In these patients the average dose of GCs was 6.7 ± 4.3 mg/day, median dose 5 mg/day, range 4-20 mg/day. 14 patients were past users of GCs and 5 patients had never used GCs.

No prevalent clinical vertebral fractures were demonstrated in the subjects under study. In the JIA patients, morphometric vertebral fractures were documented in 5 patients and non-vertebral fractures in 6 patients. No fractures were observed in the control subjects. No parental history of hip fracture was reported in subjects under study. Smoking was reported in 7 patients and alcohol abuse was not reported. No statistical differences of demographic characteristics between the patient and control group were found except for higher serum 25-hydroxyvitamin D levels in the JIA patients.

Compared to healthy controls, aBMD in JIA patients was lower at all measured sites (Table 2). In the total body as well as in the trunk and extremities (both legs and arms), no significant differences in tissue mass were observed between JIA patients and control subjects. However, in all measured regions, lean mass and BMC fraction was significantly lower, and fat mass fraction was significantly higher in JIA patients compared to controls. Chair rise test and walking speed in patients with JIA was significantly worse as compared with the control subjects (Table 2). Significant differences in body composition between JIA and controls were also evident in both genders (Table 3). BMC was reduced in all the measured sites, lean mass was reduced as well and fat mass was increased in all measured areas except for the trunk.

**Table 2:** BMD, body composition, and physical performance in JIA patients and in controls

	**JIA patients (n = 31)**	**Controls (n = 84)**	**p**
LS BMD (g/cm^2^)	1.09 ± 0.15	1.226 ± 0.10	<0.001
LS T-score	-0.54 ± 1.09	0.25 ± 0.85	0.003
Total femur BMD (g/cm^2^)	0.92 ± 0.16	1.14 ± 0.10	<0.001
Total femur T-score	-0.94 ± 1.13	0.67 ± 0.77	<0.001
Femoral neck BMD (g/cm^2^)	0.93 ± 0.17	1.13 ± 0.11	<0.001
Femoral neck T-score	-0.97 ± 1.21	0.54 ± 0.88	<0.001
Trochanter BMD (g/cm^2^)	0.69 ± 0.14	0.89 ± 0.10	<0.001
Trochanter T-score	-1.73 ± 1.35	-0.01 ± 0.97	<0.001
Distal radius BMD (g/cm^2^)	0.68 ± 0.08	0.73 ± 0.07	0.007
Distal radius T-score	-0.59 ± 1.06	-0.30 ± 0.77	0.168
Total body BMD (g/cm^2^)	1.07 ± 0.19	1.21 ± 0.08	<0.001
Total body T-score	-0.40 ± 0.92	0.64 ± 0.82	<0.001
Total body composition			
Tissue mass (g)	63914 ± 11660	66813 ± 12338	0.273
Fat mass (%)	31.67 ± 10.63	24.93 ± 8.71	0.001
Lean mass (%)	64.64 ± 10.28	70.86 ± 8.52	0.002
BMC (%)	3.70 ± 0.48	4.20 ± 0.40	<0.001
Arms composition			
Tissue mass (g)	7116 ± 1846	7446 ± 2017	0.578
Fat mass (%)	30.28 ± 12.23	21.79 ± 11.03	<0.001
Lean mass (%)	65.47 ± 11.69	73.28 ± 10.71	0.001
BMC (%)	4.25 ± 0.80	4.94 ± 0.69	<0.001
Legs composition			
Tissue mass (g)	22922 ± 4009	23974 ± 4134	0.238
Fat mass (%)	34.86 ± 12.03	26.62 ± 9.36	<0.001
Lean mass (%)	61.33 ± 11.54	68.89 ± 9.04	<0.001
BMC (%)	3.82 ± 0.62	4.49 ± 0.50	<0.001
Trunk composition			
Tissue mass (g)	30207 ± 6538	31302.0 ± 6245	0.426
Fat mass (%)	31.20 ± 11.28	25.84 ± 9.15	0.013
Lean mass (%)	66.39 ± 11.10	71.31 ± 9.03	0.021
BMC (%)	2.41 ± 0.37	2.85 ± 0.32	<0.001
Chair rise (5x) (sec)	10.29 ± 4.07	6.24 ± 1.47	<0.001
Walking rate (m/sec)	1.22 ± 0.33	1.40 ± 0.27	0.002

**Table 3:** Body composition in women and men with JIA

**Women**		**JIA (n =19)**	**Controls (n = 48)**	
	**Region**	**Mean ± SD**	**Mean ± SD**	**p**
Total body	Fat (%)	36.73 ± 8.72	29.92 ± 7.48	0.003
	Lean (%)	59.68 ± 8.37	65.88 ± 7.14	0.005
	BMC (%)	3.59 ± 0.45	4.20 ± 0.47	<0.001
Arms	Fat (%)	37.27 ± 9.46	28.64 ± 9.76	0.003
	Lean (%)	58.65 ± 8.77	66.39 ± 9.08	0.004
	BMC (%)	4.08 ± 0.92	4.97 ± 0.82	<0.001
Legs	Fat (%)	41.63 ± 9.11	32.96 ± 6.96	<0.001
	Lean (%)	54.81 ± 8.78	62.73 ± 6.64	<0.001
	BMC (%)	3.56 ± 0.53	4.30 ± 0.54	<0.001
Trunk	Fat (%)	34.98 ± 10.81	29.84 ± 8.68	0.057
	Lean (%)	62.71 ± 10.73	67.31 ± 8.51	0.084
	BMC (%)	2.31 ± 0.32	2.85 ± 0.37	<0.001
**Men**		**JIA (n = 12)**	**Controls (n = 36)**	
	**Region**	**Mean ± SD**	**Mean ± SD**	**p**
Total body	Fat (%)	24.07 ± 8.69	18.59 ± 5.46	0.015
	Lean (%)	72.07 ± 8.34	77.21 ± 5.32	0.019
	BMC (%)	3.86 ± 0.48	4.20 ± 0.29	0.006
Arms	Fat (%)	19.79 ± 7.48	13.27 ± 4.87	0.001
	Lean (%)	75.69 ± 7.16	81.83 ± 4.72	0.002
	BMC (%)	4.52 ± 0.50	4.90 ± 0.50	0.031
Legs	Fat (%)	25.56 ± 8.99	18.74 ± 4.86	0.002
	Lean (%)	70.25 ± 8.55	76.55 ± 4.71	0.003
	BMC (%)	4.20 ± 0.54	4.71 ± 0.35	<0.001
Trunk	Fat (%)	25.53 ± 9.81	20.87 ± 7.11	0.087
	Lean (%)	71.90 ± 9.58	76.27 ± 7.03	0.102
	BMC (%)	2.56 ± 0.41	2.86 ± 0.25	0.006

**Figure 1:**
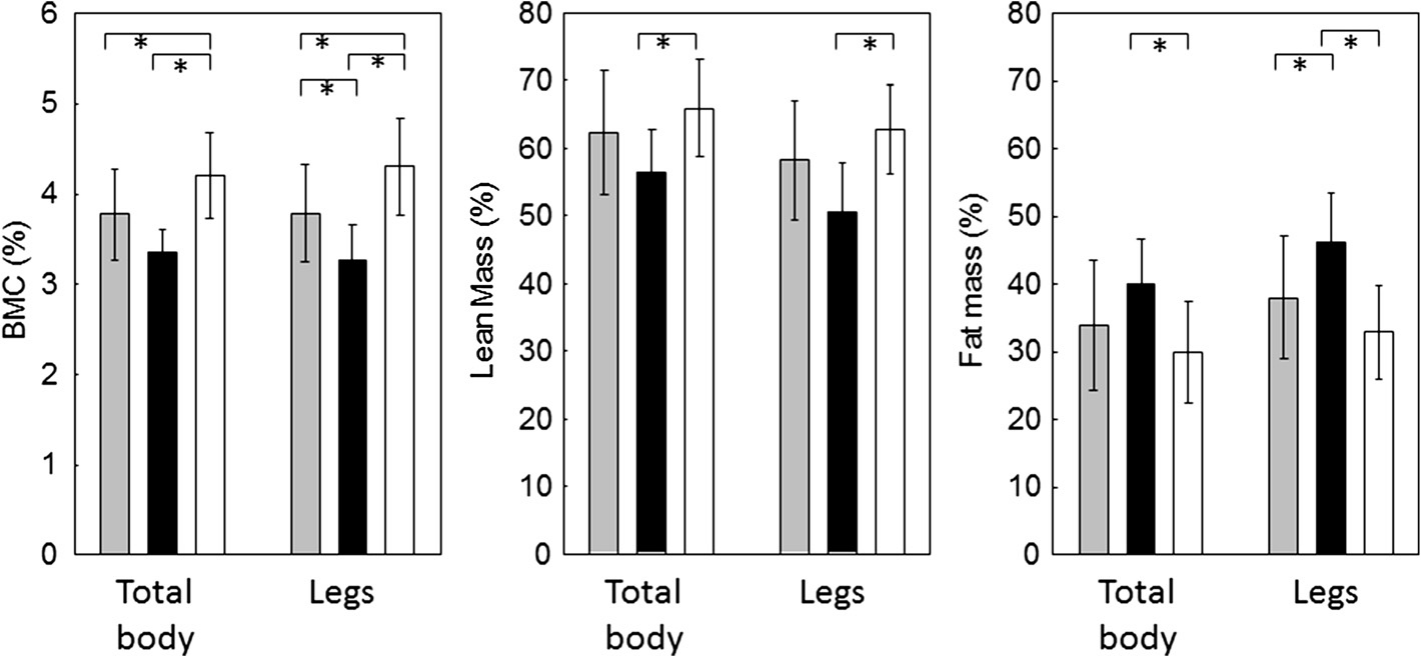
**Body composition of total body and legs in women with JIA not treated with GCs (gray bars), in women with JIA treated with GCs (black bars) and in women from the control group (empty bars).** *p < 0.05, One-way ANOVA.

In women with JIA, body composition at the total body and legs was significantly different from that in women untreated with glucocorticoids and from that in women in the control group (Figure 1).

**Figure 2:**
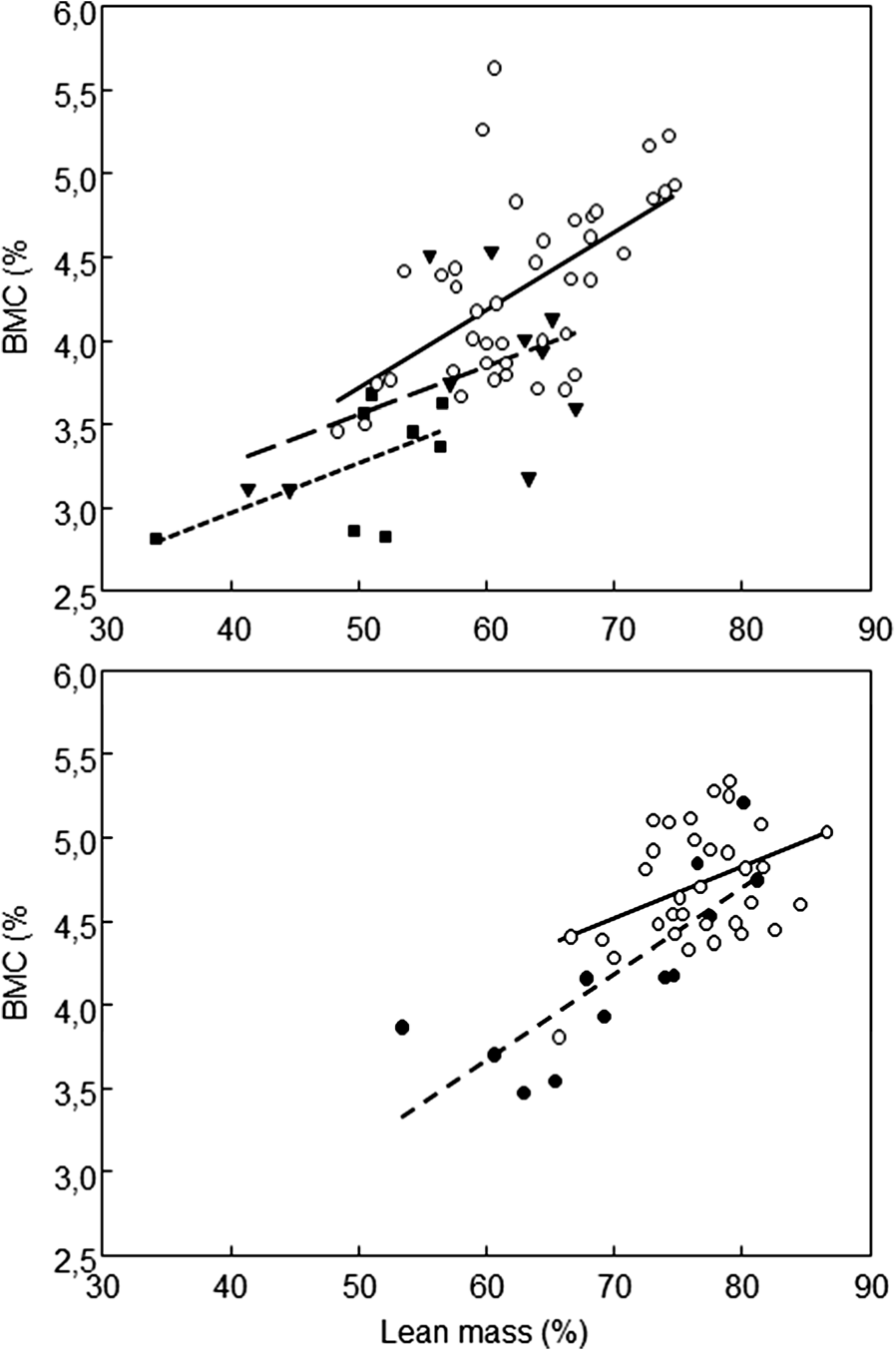
Correlations between lean tissue and BMC fractions in legs in women (upper panel) and in men (lower panel); control subjects (empty circles, full line, women, r = 0.58, p < 0.001, men, r = 0.43, p = 0.017), women with JIA not treated with glucocorticoids (full triangles, dashed line, r = 0.47, p = 0.17), women with JIA treated with glucocorticoids (full squares, dotted line, r = 0.43, p = 0.29) and men with JIA (full circles, dashed line, r = 0.81, p = 0.001).

In JIA patients, significant correlations were observed between indices of composition of legs, and physical performance and disease duration (Table 4). Significant correlations were also observed in these patients between the indices of body composition by gender (Table 5). Thanks to the inclusion of a number of female patients treated with GCs, it was possible to observe a significant negative correlation between GCs usage and BMC of legs, and between GCs usage and DAS 28. The association between lean mass and BMC in legs of women with JIA treated with GCs, not treated with GCs, and healthy women as well as in men with JIA and control subjects is given in Figure 2.

**Table 4:** Pearson correlation coefficients between body composition of legs, physical performance and disease duration in patients with JIA

	**Glucocorticoids**	**Disease duration**	**Fat tissue (%)**	**Lean tissue (%)**	**BMC (%)**	**DAS28**
Chair rise (s)	0,02	0,39*	0,40*	-0,40*	-0,23	0,13
Glucocorticoids		0,21	0,43*	-0,43*	-0,43*	0,50**
Disease duration (yrs)			0,48**	-0,47**	-0,47**	-0,04
Fat tissue (%)				-1.00**	-0,80**	0,24
Lean tissue (%)					0,78**	-0,24
BMC (%)						-0,27

*p < 0.05; **p < 0.01.

**Table 5:** Pearson correlation coefficients between composition of legs in women and men with JIA

**Women**	**Disease duration**	**Fat tissue (%)**	**Lean tissue (%)**	**BMC(%)**	**DAS28**
Glucocorticoids	0,29	0,46	-0,44	-0,50*	0,53*
Disease duration (yrs)		0,46	-0,45	-0,48*	-0,27
Fat tissue (%)			-1.00***	-0,64***	0,05
Lean tissue (%)				0,61**	-0,04
BMC (%)					-0,13
**Men**	**Disease duration**	**Fat tissue (%)**	**Lean tissue (%)**	**BMC (%)**	**DAS28**
Glucocorticoids	-0,16	0,33	-0,34	-0,23	0,32
Disease duration (years)		0,31	-0,31	-0,27	-0,02
Fat tissue (%)			-1.00***	-0,83***	-0,04
Lean tissue (%)				0,81**	0,04
BMC (%)					-0,10

*p < 0.05; **p < 0.01; ***p < 0.001.

Using multiple linear regression analysis, the fraction of BMC was significantly predicted by total body lean mass both in women (total body, p = 0.002) and in men (total body, p = 0.022, and in legs, p = 0.008), while current GC therapy, DAS28, and duration of disease did not contribute significantly to the prediction of the BMC in the patients with JIA.

## Discussion and conclusions

In our study of TNFα blocker naïve patients with JIA, a reduction of aBMD at all measured areas of the skeleton, except the distal radius T-score was observed. Decreased aBMD has been reported also in children and adolescents with JIA at all skeletal sites. Zak et al. described low bone mass density in LS spine and hips in 42-52% of adult JIA patients, both male and female [8]. In our study, a statistically significant dependence was seen between bone mass density parameters (BMD in g/cm^2^ and T-score in the area of proximal femur, femoral neck and trochanter, BMD in g/cm^2^ of distal radius and total body T-score) and the lean mass. Significant correlations were observed between BMC and disease duration, GCs usage and lean mass, respectively. Lean mass was the only determining factor of total proximal femur BMD and femoral neck BMD. Also, lean mass was the main determinant of the total body BMC both in women and men, and also of the leg BMC in men. This is in good agreement with the observations on body composition in non-corticosteroid–treated postpubertal women and in prepubertal children with JIA [15-17].

The differences between body composition of total body and legs in the subgroup of women with JIA treated and not treated with GCs indicate a negative effect of GCs on the lean mass and BMC, and the positive effect on fat tissue. This is in good agreement with several cross-sectional and longitudinal studies demonstrating substantial effects of GCs on muscle atrophy and body composition in patients with medical illnesses such as Crohn’s disease, multiple sclerosis, systemic lupus erythematosus, glucocorticoid-sensitive nephrotic syndrome and post-renal transplantation [18-23]. The significant positive correlation between the activity of the disease and GC use could be explained by the necessity of GC therapy in patients with severe course of disease. However, while 9 out of 19 women patients were on GC therapy, the BMC fraction was significantly predicted by GC use rather than by DAS28. The importance of lean mass is further supported by the significant correlation between disease duration and increase of fat mass and reduction of bone and lean mass and deteriorated physical performance of legs evaluated using the chair test. In a study where lean mass and cortical and trabecular bone forearm BMD were measured using peripheral quantitative computed tomography, JIA patients had significantly reduced muscle cross-sectional area and this reduction significantly correlated with muscle strength and bone geometry abnormalities and, particularly, with reduced thickness of the cortical bone [24]. Similar conclusions were derived from the measurement of muscle and bone mass of the tibia [25]. Prolonged exposure to GCs can lead to muscle atrophy.

The aforementioned results support the hypothesis that muscles (at least in adults) play a dominant role in the synchronization of muscle and bone mass [26]. This closely linked function and form of both tissues may be, from the embryonic development to the old age, influenced by genetic dispositions, morphogenic factors, sex steroids and, in adulthood, particularly mechanical signals [27], inter alia through myokines (myostatin, leukemia inhibitory factor, interleukin 6, interleukin 7, insulin-like growth factor 1, fibroblast growth factor 2, follistatin-like protein and irisin) [28]. The myostatin/activin signaling pathway may be involved in both muscle and bone coordination [29]. Increased cytokine production during long-lasting inflammations induces protein degradation, inhibits myocyte differentiation and induces apoptosis of myocytes and myopathy [30]. In JIA, inflammation may be, through muscular mass reduction, responsible also for reduced bone mass. As muscles are the main source of myostatin and the administration of glucocorticoids is associated with an increased production of myostatin, muscular atrophy and increased secretion of myostatin in active JIA further suppresses new bone formation and induces reduction of BMD [31,32]. The cause of the myopathic condition is not necessarily limited to the inflammatory cytokines – it could also involve GCs and the lower physical activity in JIA patients [24,30]. Glucocorticoids not only decrease muscle anabolism by inhibiting amino acid transport into the muscle [8], but also increase muscle catabolism [33]. GCs play a key role in inducing proteolysis in acute inflammatory states via the autophagy and the ubiquitin–proteasome pathways [34].

Several limitations of the study must be taken into account. First, the sample size was not large enough to make definite conclusions by multiple logistic regression analysis. Secondly, the results do not allow for an assessment of the association of changes in tissue composition with the risk of fracture [35]. The established correlations between mass and bone unit may be influenced by genetic factors [36] and individual differences in physical activity and diet [37,38] that were not controlled in this study. Also, individual patient susceptibility to adverse effects of GCs depends on GC dose, duration of therapy, GC receptor saturation levels and GC receptor gene polymorphisms [33]. The cross-sectional nature of the study does not allow for a more accurate assessment of the muscular-bone unit relationship in individual patients and the disease activity.

The results of this study show significant effects of both the disease and GC therapy on aBMD and body composition in patients with JIA and support the hypothesis of the dominant role of muscles in the synchronization of muscular and bone mass.

## Competing interest

The authors stated that there are no conflicts of interest regarding the publication of this article. The manuscript has not been submitted or published elsewhere.

## Authors’ contribution

KBM carried out the laboratory tests, participated in the examination of the patients and the healthy subjects and drafted the manuscript. KJ participated in the clinical examination of the patients. KP participated in the writing the manuscript. JJS participated in the study design, data analysis, data interpretation and writing the manuscript. All authors read and approved the final manuscript.

## Pre-publication history

The pre-publication history for this paper can be accessed here:

http://www.biomedcentral.com/1471-2474/15/51/prepub
